# Sensitivity of ocean circulation to warming during the Early Eocene greenhouse

**DOI:** 10.1073/pnas.2311980121

**Published:** 2024-06-03

**Authors:** Sandra Kirtland Turner, Andy Ridgwell, Allison L. Keller, Maximilian Vahlenkamp, Adam K. Aleksinski, Philip F. Sexton, Donald E. Penman, Pincelli M. Hull, Richard D. Norris

**Affiliations:** ^a^Department of Earth and Planetary Sciences, University of California, Riverside, CA 92521; ^b^Center for Marine Environmental Sciences (MARUM), University of Bremen, Bremen 28359, Germany; ^c^Department of Earth, Atmospheric and Planetary Sciences, Purdue University, West Lafayette, IN 47906; ^d^School of Environment, Earth and Ecosystem Sciences, The Open University, Milton Keynes MK7 6AA, United Kingdom; ^e^Department of Geosciences, Utah State University, Logan, UT 84322; ^f^Department of Earth and Planetary Sciences, Yale University, New Haven, CT 06511; ^g^Center for Marine Biodiversity and Conservation, Scripps Institution of Oceanography, University of California San Diego, La Jolla, CA 92093

**Keywords:** hyperthermals, ocean overturning circulation, paleoceanography, Earth system modeling

## Abstract

Multiple abrupt warming events (“hyperthermals”) punctuated the Early Eocene and were associated with deep-sea temperature increases of 2 to 4 °C, seafloor carbonate dissolution, and negative carbon isotope (δ^13^C) excursions. Whether hyperthermals were associated with changes in the global ocean overturning circulation is important for understanding their driving mechanisms and feedbacks and for gaining insight into the circulation’s sensitivity to climatic warming. Here, we present high-resolution benthic foraminiferal stable isotope records (δ^13^C and δ^18^O) throughout the Early Eocene Climate Optimum (~53.26 to 49.14 Ma) from the deep equatorial and North Atlantic. Combined with existing records from the South Atlantic and Pacific, these indicate consistently amplified δ^13^C excursion sizes during hyperthermals in the deep equatorial Atlantic. We compare these observations with results from an intermediate complexity Earth system model to demonstrate that this spatial pattern of δ^13^C excursion size is a predictable consequence of global warming-induced changes in ocean overturning circulation. In our model, transient warming drives the weakening of Southern Ocean-sourced overturning circulation, strengthens Atlantic meridional water mass aging gradients, and amplifies the magnitude of negative δ^13^C excursions in the equatorial to North Atlantic. Based on model-data consistency, we conclude that Eocene hyperthermals coincided with repeated weakening of the global overturning circulation. Not accounting for ocean circulation impacts on δ^13^C excursions will lead to incorrect estimates of the magnitude of carbon release driving hyperthermals. Our finding of weakening overturning in response to past transient climatic warming is consistent with predictions of declining Atlantic Ocean overturning strength in our warm future.

## Significance

Numerous transient, greenhouse-gas-fueled warming events called “hyperthermals” occurred during the Early Eocene Climate Optimum (~53.26 to 49.14 Ma). The extent of warming and carbon cycle perturbation across hyperthermals are constrained primarily by benthic foraminiferal stable isotope records. Here, we show spatial patterns in the magnitude of the negative carbon isotope excursions that characterize these events. Using an intermediate complexity Earth system model, we demonstrate that this pattern indicates transient weakening of the large-scale ocean overturning circulation and enhanced deep ocean aging gradients coincident with hyperthermal warming. Our results show that globally distributed paleoceanographic records that capture gradients in deep water mass properties are critical to accurately constrain carbon forcing across past transient climatic events.

Changes in the ocean’s large-scale, density-driven circulation have been linked to a variety of prominent past climatic transitions and perturbations—from the glacial–interglacial cycles of the Plio-Pleistocene (last ~3 My) (1–6) to transient “hyperthermals” during the Early Eocene (~56 to 48 Ma) (7–10) and to the long-term evolution of ocean oxygenation during the Phanerozoic (11). By modulating the strength of the biological and solubility carbon “pumps” in the ocean, changes in global overturning circulation impact atmospheric *p*CO_2_ (1, 12–15). Furthermore, changes in large-scale circulation and redistribution of heat in the ocean interior across hyperthermals could also have indirectly contributed to changing *p*CO_2_ by destabilizing subseafloor methane hydrates (8, 16, 17). Ocean circulation may thus act as a powerful feedback mechanism, linking atmospheric *p*CO_2_ to climate change and operating across a wide range of background climate states, from “ice-house” to “greenhouse.”

In the modern ocean, North Atlantic Deep Water (NADW) carries a positive δ^13^C signature of nutrient-depleted surface water to depth, which contrasts with the slightly more negative δ^13^C and more nutrient-replete surface signature of the Southern Ocean that is carried northward at depth by Antarctic Bottom Water (AABW) (1). Both contrast with the older, more poorly ventilated waters of the deep North Pacific that are rich in nutrients and respired carbon and thus exhibit very low δ^13^C (18). The spatial patterns of benthic δ^13^C, recorded in the carbonate tests of benthic foraminifera, hence represent a potential means of constraining past geometries of the large-scale overturning circulation. Indeed, analysis of benthic δ^13^C gradients has been the foundation of discoveries of a glacial-age shoaling of NADW (forming Glacial North Atlantic Intermediate Water) and an extension of AABW into the North Atlantic, far north of its interglacial position (1, 15, 19).

In the acute warmth of the early Eocene, ocean overturning circulation appears to have been driven by deepwater formation in the Atlantic and Indian sectors of the Southern Ocean, indicated by sites in these regions consistently yielding the highest benthic foraminiferal δ^13^C values (20, 21). Nd isotope data—an alternative proxy for water mass source and flow pathways (22, 23)—also supports the inference of a Southern Ocean dominance of overturning during the Eocene (24), with additional possible sources of deepwater formation located in the North Pacific (25–29). In contrast, based on benthic foraminiferal δ^13^C, the Eocene North Atlantic does not appear to be proximal to any source of deepwater (20, 21, 24, 30).

Climate model experiments suggest that the large-scale overturning circulation in an Eocene greenhouse climate may have been particularly sensitive to orbital forcing of insolation (17, 31). This is supported by geological data indicating changing interbasin δ^13^C gradients across the two most prominent hyperthermals in the Early Eocene, the Paleocene Eocene Thermal Maximum (~56 Ma), and Eocene Thermal Maximum 2 (ETM-2, ~54 Ma) (7, 8). Numerous other orbitally paced hyperthermal events exist in the early Eocene (32–36). However, to date, no equivalent evidence exists for changes occurring in overturning circulation across these other hyperthermals, owing to the scarcity and low-resolution of existing records and the challenges in creating the sufficiently well-constrained age models needed to make the necessary precise intersite correlations.

Here, we present two high-resolution benthic foraminiferal stable isotope datasets from the equatorial Atlantic (ODP Site 1258) and North Atlantic (IODP Site U1409) throughout the Early Eocene Climate Optimum (EECO) that reveal numerous previously identified hyperthermals in the form of paired negative excursions in δ^13^C and δ^18^O. We compare these records with previously published high-resolution datasets from the South Atlantic (ODP Site 1263) and central Pacific (ODP Site 1209) that cover the same time interval. We further evaluate the potential impact and signal of greenhouse-gas-driven warming on the ocean overturning circulation using the intermediate complexity Earth system model cGENIE (37, 38). Specifically, we investigate how changes in ocean circulation associated with global warming are imprinted on typical model metrics of overturning (e.g., the overturning stream function) as well as on spatial patterns in deep water age and the δ^13^C of dissolved inorganic carbon (DIC) (comparable to our benthic foraminiferal data and hence providing model-derived predictions to validate our observational evidence). We find amplification of δ^13^C excursions in the equatorial Atlantic in both data and model experiments, which we interpret as evidence that Early Eocene hyperthermals were characterized by transient alternations in the strength and pattern of ocean overturning circulation.

## 1. Materials and Methods

ODP Site 1258 is located on Demerara Rise in the equatorial Atlantic with an Eocene paleodepth of ~3,000 m (21). Site 1258 contains a complete record of foraminifer nannofossil chalks of ~60 wt% CaCO_3_ across the EECO interval (21). Ref. 32 previously reported high-resolution bulk carbonate δ^13^C and δ^18^O at 10-cm spacing from 56 to 131 revised meters composite depth (mcd) to reveal the existence of multiple orbitally paced hyperthermal events. Here, we sampled the same interval in order to obtain a comparable benthic foraminiferal dataset at 10-cm spacing, aligning samples with the bulk carbonate-based dataset as much as possible. Based on the astronomically tuned age model for Site 1258 from ref. 39, the benthic foraminiferal dataset has average sample resolution of ~6 ky.

IODP Site U1409 is located on the Southeast Newfoundland Rise in the North Atlantic and also has a reconstructed Eocene paleodepth of ~3,000 m (40). The EECO interval was identified by shipboard biomagnetostratigraphy as a nannofossil ooze with ~90 wt% CaCO_3_ between ~123 and 173 mcd; however, incomplete recovery due to the presence of intermittent cherts during drilling prevented continuous coverage through the EECO interval (40). We selected samples at 5-cm spacing in order to generate a benthic foraminiferal dataset with a temporal resolution of ~6 ky based on the shipboard age model.

Samples from both sites were disaggregated and washed in deionized water over a 63 μm mesh sieve and specimens of the benthic foraminifer *Nuttalides truempyi* were picked from the >150 μm size fraction. The final datasets contain 751 benthic foraminiferal measurements from Site 1258 and 914 from Site U1409. The earliest interval of EECO samples from Site 1258 contained a number of samples where benthic foraminifera were rare, so we have no corresponding benthic foraminiferal analyses to the original bulk carbonate stable isotope record from these samples.

All stable isotope measurements were conducted using a Kiel IV carbonate device coupled to a Delta V isotope ratio mass spectrometer using standard dual inlet techniques at the University of California, Riverside. The long-term reproducibility based on replicate analyses of an in-house standard run concurrently with unknown samples was <0.03‰ for δ^13^C and <0.07‰ for δ^18^O. All values are reported relative to the Vienna Peedee Belemnite (VPDB) standard.

All data are reported against astronomically tuned age models consistent with ref. 35. For ODP Site 1258, we used the age model of ref. 39, that has also been used in tuning the ODP Site 1209 benthic foraminiferal stable isotope record across the Paleocene to middle Eocene (35) (Dataset S1). For Site U1409, we tuned bulk δ^13^C and δ^18^O to the astronomically tuned age models for Site 1258, 1262, and 1263 used in ref. 35 (Dataset S2; tie points are provided in Dataset S3).

**Fig. 1.**
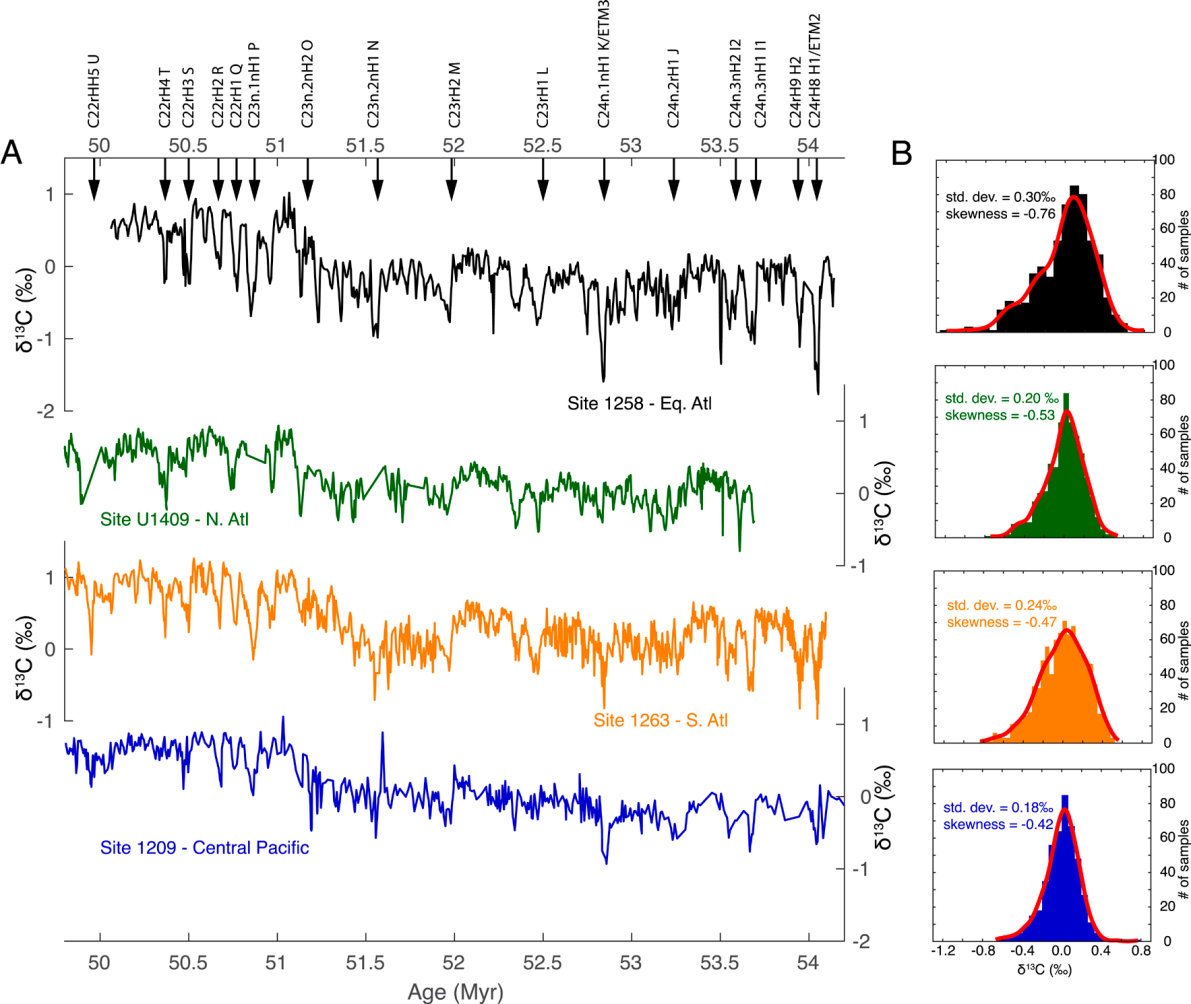
(*A*) Benthic foraminiferal δ^13^C records from four sites across the EECO, from *Top* to *Bottom*: ODP Site 1258 in the equatorial Atlantic, IODP Site U1409 in the North Atlantic, ODP Site 1263 in the South Atlantic, and ODP Site 1209 in the Central Pacific. Labeled arrows indicate hyperthermal events identified from Site 1209 by ref. 35. (*B*) Kernel fit distribution and statistics (SD and skewness) of detrended benthic foraminiferal δ^13^C data across the EECO at each of four sites.

We used the intermediate complexity Earth system model cGENIE to evaluate the impact of hyperthermal warming on ocean overturning circulation. The cGENIE model includes a 3-D dynamic ocean model with a simplified energy and moisture balance atmosphere (41) and a representation of the biogeochemical cycling of elements and isotopes (42). Model resolution is 36 × 36 × 16 with grid spacing that is equal in longitude and the sine of latitude and exponentially increasing grid thickness with ocean depth (*SI Appendix*, Fig. S1). This model is capable of capturing the large-scale structure of modern ocean circulation and its attendant δ^13^C patterns (38, 43). We used the early Eocene configuration of ref. 44, with a 10 ky spin-up to equilibrate the large-scale ocean circulation. In this configuration, global overturning is dominated by counterclockwise meridional overturning circulation, driven by deepwater formation in the Southern Ocean in South Atlantic and South Pacific (*SI Appendix*, Figs. S2–S6). Consequently, the modeled δ^13^C of DIC in the deep ocean matches the broad pattern of early Paleogene benthic δ^13^C data, with the highest values in the Southern Ocean consistent with newly formed deep water (*SI Appendix*, Figs. S3 and S4) (8, 21, 32). The modeled overturning circulation is similar to fully coupled Eocene climate models (24). We modeled a generic early Eocene hyperthermal by forcing model atmospheric CO_2_ δ^13^C to follow an excursion of −1 ‰ over 10 ky, with a full recovery after 40 ky, by adding and removing CO_2_ from the atmosphere with an isotopic composition of −25‰. The model achieves this with total carbon input of just over 1,500 Pg C and a maximum addition rate of <0.2 Pg C y^−1^. Over the modeled early Eocene hyperthermal, atmospheric CO_2_ increases from 834 ppm (3× preindustrial) to ~1,300 ppm and atmospheric temperature rises consequently by ~2 °C. The model experiment hence reproduces a relatively modest carbon cycle perturbation and warming event, consistent with the available benthic δ^13^C and δ^18^O data (Fig. 1 and *SI Appendix*, Fig. S7).

## 2. Results

Our benthic stable isotope records from Site 1258 in the equatorial Atlantic and Site U1409 in the North Atlantic encompass nearly the entirety of the EECO (from 53.26 to 49.14 Ma according to ref. 35) (Fig. 1 and *SI Appendix*, Fig. S7). Records from both sites reproduce both long-term trends and short-term events revealed by existing high-resolution records across this time interval from ODP Site 1209 in the central Pacific and ODP Site 1263 in the South Atlantic (35). At all sites, long-term δ^18^O values begin to decrease at ~52 Ma, reaching a minimum of nearly −1.5‰ at Site 1258, indicative of maximum deep water temperatures at ~51.5 Ma, and followed by a ~0.5 My interval of relative cooling. These trends are also visible in the Site U1409 benthic δ^18^O record, though the minimum δ^18^O is slightly higher (~−1.3‰) and the cooling interval at ~51 Ma is less pronounced. The cooling event at ~51 Ma is significantly less apparent at Sites 1263 and Site 1209. The ~51 Ma δ^18^O increase apparent at Sites 1258 and U1409 also coincides with a pronounced positive shift in benthic δ^13^C. This shift in δ^13^C was previously identified both in the Site 1258 bulk carbonate record (32) and in benthic foraminiferal datasets (35) but previously was not clearly associated with evidence for cooling.

Hyperthermal events identified across EECO at multiple sites by ref. 35 are marked by distinct correlative negative excursions in benthic δ^13^C and δ^18^O at Site 1258 (Fig. 1). The Site U1409 record from the North Atlantic also reveals paired negative excursions in benthic δ^13^C and δ^18^O across most of the identified EECO hyperthermals, despite the impact of intermittent cherts on the completeness of the record (39). However, δ^13^C excursions (and to a lesser extent δ^18^O excursions) at Site 1258 in the equatorial Atlantic are clearly amplified relative to the other sites (Fig. 1). We characterize the relative magnitude of hyperthermals across sites by evenly resampling and detrending each δ^13^C record and comparing the distribution of δ^13^C measurements, SD of each dataset, and skewness. All sites show a negative skew in their δ^13^C distribution, but the detrended δ^13^C record from Site 1258 has the greatest range, the largest SD, and the largest (negative) skewness compared to the other three sites, consistent with larger magnitude δ^13^C excursions (Fig. 1). In comparison, the range, SD, and skewness from the other three sites are more similar.

**Fig. 2.**
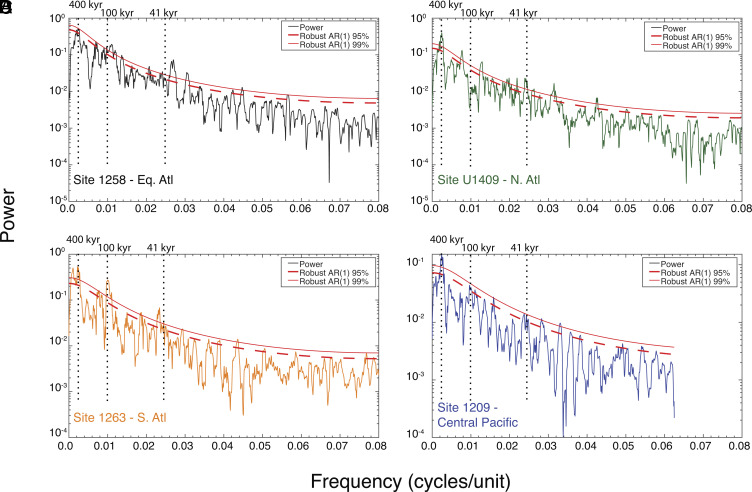
MTM-power spectra of benthic foraminiferal δ^13^C records across the EECO (*A*) Site 1258, (*B*) Site U1409, (*C*) Site 1263, and (*D*) Site 1209.

To further assess the character of δ^13^C excursions throughout each record, we compare Multi-Taper Method (MTM) power spectra for each evenly sampled and detrended timeseries (Fig. 2) (45). Previous research has consistently indicated that Early Eocene hyperthermals are paced by variations in long and short eccentricity (33–36, 46–49). While we find high power at the long eccentricity period in the δ^13^C records from each site, and significant power at the short eccentricity period for all sites except Site U1409 in the North Atlantic, we also find significant power near the obliquity period at all sites except the lower resolution Site 1209 record in the North Pacific (35). This result is consistent with the relatively shorter duration of hyperthermal events from ~51.5 to 50 Ma, corresponding to the prominent positive shift in δ^13^C from ~51.5 to 51 Ma. The finding of obliquity pacing between ~52 and 50 Ma was previously identified in XRF-derived Fe intensity records from Site 1258 (39).

**Fig. 3.**
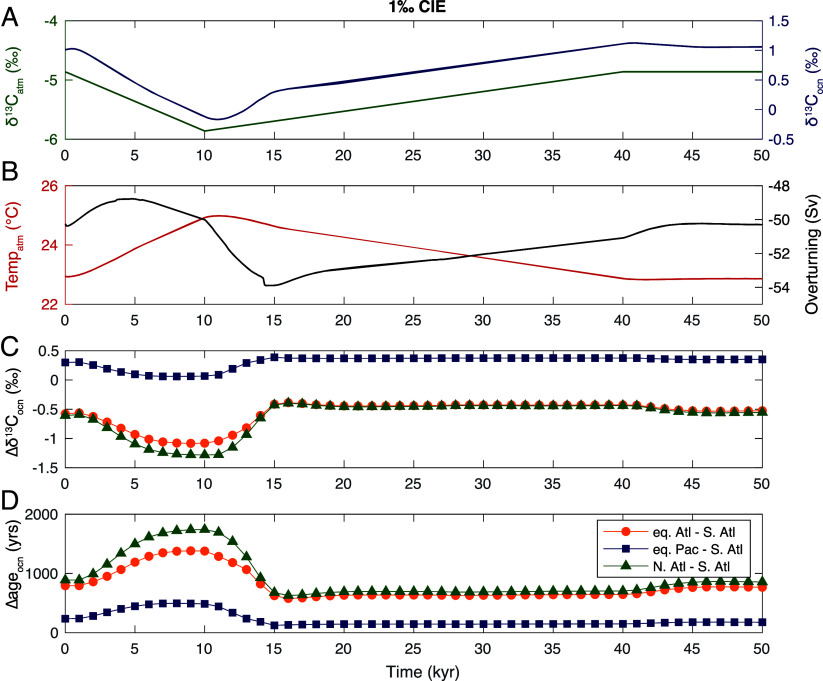
cGENIE model output of changes in climate and ocean circulation in response to a −1‰ CIE. (*A*) Atmospheric δ^13^C (green) and benthic ocean DIC δ^13^C (blue). (*B*) Atmospheric temperature (red) and global counterclockwise overturning streamfunction (black). (*C*) Change in the deep ocean DIC δ^13^C gradient between the equatorial Atlantic and South Atlantic (orange), the North Atlantic and South Atlantic (green), and between the equatorial Pacific and South Atlantic (blue). (*D*) Changes in water mass aging gradients between the equatorial Atlantic and South Atlantic (orange), the North Atlantic and South Atlantic (green), and between the equatorial Pacific and South Atlantic (blue). Spatially resolved model output from the ocean grid (*C* and *D*) was saved at lower-resolution in comparison to global average time series (*A* and *B*) and is plotted as discrete symbols.

In our model-simulated hyperthermal, forcing the δ^13^C of atmospheric CO_2_ to follow a −1‰ excursion over 10 ky results in an average benthic oceanic DIC δ^13^C excursion of ~−1.2‰ that is delayed relative to the atmosphere by ~1 ky (Fig. 3*A*). Atmospheric CO_2_ peaks at 10.8 ky and atmospheric temperature peaks shortly after at 11 ky (Fig. 3*B*). Counterclockwise (driven by sinking in the Southern Ocean) overturning decreases over 5 ky (from ~−50.3 Sv to ~−48.8 Sv) in response to warming before rebounding by the time that atmospheric temperature peaks and subsequently overshooting to stronger overturning (~−54 Sv) at ~14 ky (Fig. 3*B*).

We assess the impact of modeled changes in overturning circulation on δ^13^C and water mass age at model locations corresponding to each of our four sites by calculating gradients between each location throughout the simulated hyperthermal (Fig. 3 *C* and *D*). The δ^13^C gradient between the central Pacific and South Atlantic (blue line, Fig. 3*C*) decreases slightly while the water mass age gradient between these two locations increases slightly (blue line, Fig. 3*D*). In contrast, the magnitude of gradients in δ^13^C and water mass age both increase substantially between the equatorial Atlantic and South Atlantic (orange lines, Fig. 3 *C* and *D*) and between the North Atlantic and South Atlantic (green lines, Fig. 3 *C* and *D*). The changing gradients in age and δ^13^C are consistent with changes in the relative contributions of different deep ocean water masses at each location in response to ocean warming and changing overturning strength. However, the minimum of the streamfunction (Fig. 3*B*) does not correspond temporally to the maximum in age gradients (Fig. 3*D*). The maximum in aging gradients varies in both magnitude and timing as a function of ocean depth, with smaller increases in ocean age that reach a maximum slightly earlier at shallower ocean depths. The age gradient between the equatorial Pacific and South Atlantic (Fig. 3*D*, blue) reaches its relative maximum earlier than the age gradient between the equatorial Atlantic or North Atlantic and South Atlantic (Fig. 3*D*, orange & green), consistent with the deeper model depths corresponding to the equatorial and North Atlantic sites compared to the equatorial Pacific and South Atlantic.

**Fig. 4.**
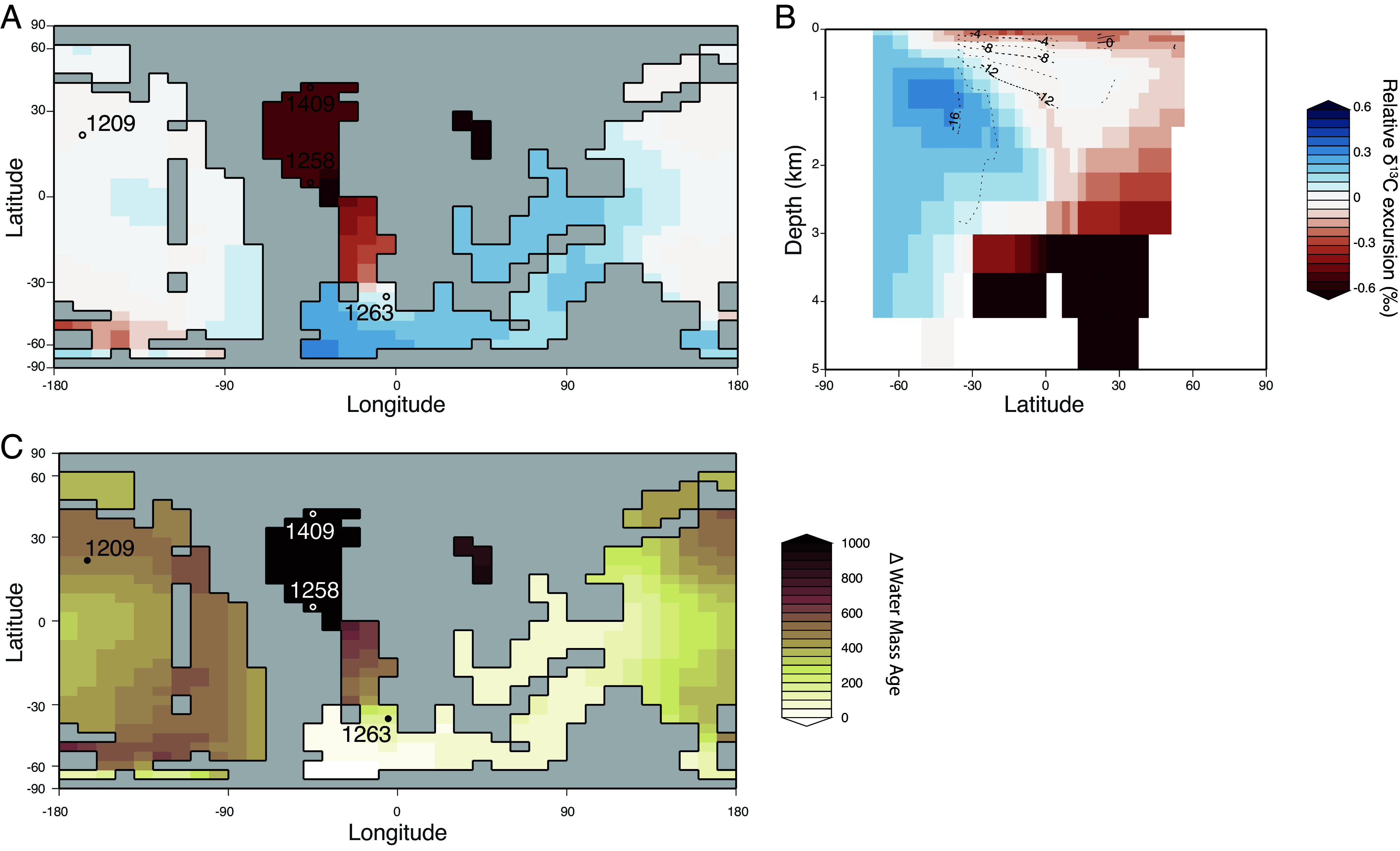
Spatial patterns in cGENIE DIC δ^13^C excursion size and water mass age in response to −1‰ CIE. (*A*) Relative size of the δ^13^C excursion recorded for each model grid relative to the mean δ^13^C excursion in the ocean layer centered at ~3,300 m water depth. δ^13^C excursions are calculated as the DIC δ^13^C value at 11 ky less the initial value in that grid. Red colors (more negative) indicate CIEs larger than the average. (*B*) Atlantic zonal mean size of the δ^13^C excursion relative to the atmospheric δ^13^C excursion. δ^13^C excursions are calculated as the DIC δ^13^C value at 11 ky less the initial value. Contours show the Atlantic Meridional Overturning streamfunction at 11 ky calculated using the mask in *SI Appendix*, Fig. S1 (for comparison to the global streamfunction, see *SI Appendix*, Fig. S2). (*C*) Relative size of the change in water mass age recorded for each model grid relative to the mean change in water mass age in the ocean layer centered at ~3,300 m water depth. Changes in water mass age are calculated as the water mass age at 11 ky less the initial age in that grid. Red colors indicate larger increases in water mass age than the global average.

We show that these changes in gradients between individual sites are consistent with spatial patterns in oceanic δ^13^C excursion size by calculating the relative size of the DIC δ^13^C excursion compared to the global average (Fig. 4). Across a constant depth surface—here taken to be 3,300 m and consistent with the paleodepth of equatorial Atlantic Site 1258 and North Atlantic Site U1409—the relative change in δ^13^C (or the magnitude of the δ^13^C excursion compared to the average δ^13^C excursion at that depth) shows clear spatial patterns (Fig. 4*A*), with the largest excursions preserved in the deep equatorial to northern Atlantic. Comparison of the ocean δ^13^C excursion magnitude relative to the atmosphere (Fig. 4*B*) also indicates the greatest amplification of δ^13^C excursions occurs in the deep North Atlantic. Deep equatorial to North Atlantic excursions are up to 0.6‰ larger than the modeled δ^13^C excursion in the atmosphere (an increase of more than 50%).

Spatial patterns in the size of the δ^13^C excursion are mirrored in patterns of changes in water mass age at 11 ky, when temperature increase is greatest (Fig. 3*D*). In response to carbon forcing, warming, and weaker overturning circulation, the idealized water mass ventilation age across the 3,300 m depth surface increases by up to ~1 ky in the equatorial to North Atlantic region, compared to a global average increase of ~400 y (Fig. 4*C*). These large changes in water mass age correspond to a relatively small change in overturning strength (a weakening of a few Sv). Combined with spatial and temporal variability in ocean aging gradients, this demonstrates that the streamfunction is not directly representative of changes in water mass distributions.

## 3. Discussion

Eocene hyperthermal events are identified by global recognition and correlation of negative δ^13^C excursions, while the associated carbon forcing (and hence peak change in atmospheric *p*CO_2_) is commonly determined from the magnitude of the δ^13^C excursion by using isotope mass balance and assuming the isotopic composition of the carbon source (32, 34, 35, 49). Combined with proxy constraints on temperature change, the magnitude of the δ^13^C excursion is hence important for constraining climate sensitivity. Spatial variations in δ^13^C excursion size complicate the determination of the magnitude of the global δ^13^C excursion (e.g., ref. 50). If changes in ocean circulation can amplify the largest preserved δ^13^C excursions, it will be necessary to correct δ^13^C records for this bias before they are used to constrain carbon forcing.

In order to isolate the impact of warming and changes in ocean circulation on spatial patterns in δ^13^C excursion size, we conducted a series of additional experiments in cGENIE. First, we repeated the same carbon forcing experiment, with input of −25‰ carbon in order to force the atmosphere to follow a −1‰ excursion, but excluded CO_2_-climate feedback, such that changes in *p*CO_2_ drive no changes in climate (*SI Appendix*, Figs. S8 and S10). Our results confirm that without warming (and hence without a change in overturning circulation), there are no spatial patterns in δ^13^C excursion size or ocean age due to CO_2_ injection (note the absence of spatial patterns in *SI Appendix*, Fig. S8). Second, we diagnosed the radiative forcing from our original carbon forcing experiment and imposed this radiative forcing to the atmosphere without a change in CO_2_ emissions (*SI Appendix*, Figs. S9 and S11). In this experiment, we reproduce nearly identical patterns in the relative change in deep ocean δ^13^C compared to the original experiment, with relative decreases in the δ^13^C of the equatorial to North Atlantic consistent with water mass aging (*SI Appendix*, Fig. S9). Combined, these sensitivity experiments demonstrate that the modeled patterns in δ^13^C excursion size reflect the impact of warming and weaker overturning circulation and are not a consequence of changes in ocean chemistry or preferential invasion paths of the injected isotopically depleted CO_2_ into the ocean.

In all of our model experiments, changes in export production are tied to changes in circulation (*SI Appendix*, Fig. S12). However, spatial differences in the modeled change in export production or the transfer of isotopically depleted carbon to the deep sea via the biological pump do not explain the modeled patterns in deep ocean δ^13^C excursion size (*SI Appendix*, Fig. S12). Coincident with the maximum change in temperature and the deep ocean δ^13^C excursion, export production has decreased globally and at locations corresponding to our sites in the equatorial Atlantic, North Atlantic, and North Pacific but increased in the Southern Ocean. Patterns of the δ^13^C of carbon export mirror changes in the surface ocean DIC pool. These also show a consistent change in δ^13^C between the equatorial Atlantic, North Atlantic, and North Pacific, but a relatively smaller change in the Southern Ocean. Hence, these patterns are inconsistent with amplified excursions in the equatorial and North Atlantic and indicate that changes in export production are not responsible for the modeled spatial patterns we observe.

The similarity between our model results and benthic foraminiferal δ^13^C records demonstrates that intersite differences in δ^13^C excursion size can be explained by transient alternations in the strength and pattern of ocean overturning circulation associated with hyperthermal warming. While this impact on benthic δ^13^C records complicates the determination of the global δ^13^C excursion size, persistent relationships in the relative magnitude of benthic δ^13^C excursions at individual sites offer a unique method to test for past changes in the strength of the large-scale ocean circulation. Comparing the relative size of δ^13^C excursions, as opposed to evaluating the evolving intersite δ^13^C gradients throughout individual events, has the additional benefit that it does not require precise alignment of age models between sites across each event. While the modeled changes in circulation strength are transient, recovering as surface warming propagates through the water column, proxy records of δ^13^C will record spatial patterns consistent with enhanced water mass aging along deepwater flow paths. The largest δ^13^C excursions will be preserved far from sites of new deep water formation where a loss of young, high δ^13^C waters amplifies the excursion relative to the global average.

While preservational factors might also contribute to variation in the intersite magnitude of recorded δ^13^C excursions, either amplifying or diminishing the patterns caused by changes in ocean circulation (e.g., ref. 37), it is unlikely that these overwhelm the signal caused by changes in ocean circulation. First, differences in sedimentation rate between sites cannot explain the relative size of recorded δ^13^C excursions because calculated sedimentation rates are lower at Site 1258 (~1.3 cm/ky) than at Site 1263 (~2.5 cm/ky). Larger δ^13^C excursions are expected at sites with higher sedimentation rates and hence less impact of bioturbational smoothing (37). Thus, if minimal physical mixing explained the amplification of δ^13^C excursions at Site 1258, then Site 1258 sedimentation rates should be higher than at any of the other sites across the EECO interval. Low sedimentation rates at Site 1209 (~0.6 cm/ky), however, may explain why this site has the smallest SD in the detrended δ^13^C record from this interval as well as a reduction of spectral power at higher frequencies consistent with smaller magnitude δ^13^C excursions.

Second, stratigraphic incompleteness is likely only a factor influencing the relative size of excursions at Site U1409 in the North Atlantic. The occurrence of intermittent cherts throughout the section (39), often associated with hyperthermal events (9), prevented continuous core recovery. It is likely that lower recovery reduced the SD in the detrended δ^13^C record by truncating δ^13^C excursions and reducing the total number of hyperthermals recorded. The relative incompleteness of the North Atlantic record is the most likely explanation for why δ^13^C excursions are not similarly amplified at this location compared to the equatorial Atlantic, as predicted by cGENIE.

Third, chemical erosion may have differentially impacted the records from each site, potentially truncating the magnitude of the recorded δ^13^C excursions at some sites more than others. Dissolution layers, or reductions in wt% CaCO_3_ due to ocean acidification and shoaling of the carbonate compensation depth, characteristically mark EECO hyperthermal events (35, 49). However, Site 1258 is relatively deeper and closer to the lysocline compared to the other three sites, with lower wt% CaCO_3_ throughout the EECO interval (51), so it is highly unlikely that δ^13^C excursions are less influenced by dissolution at Site 1258 in comparison to the other records.

Finally, differences in recrystallization intensity are an unlikely explanation for intersite differences in excursion size. Benthic foraminifera show similar preservation at all four sites (21, 35, 39, 52). For diagenesis to have impacted the relative size of isotopic excursions, as opposed to shifting the absolute values of the entire isotope record, recrystallization would have had to increase by relatively more across each isotopic excursion at Site 1258 in comparison to other sites. However, even if this were the case, previous studies indicate that large intersite differences in the preservation of benthic foraminiferal calcite correspond to only negligible intersite offsets in benthic δ^13^C and δ^18^O values (53).

As to where the isotopically depleted carbon “comes from,” while there is no consensus as to the cause of the Early Eocene hyperthermals, there is agreement that the events are predominantly paced by orbital variations in eccentricity and that they must be linked to periodic carbon release from a reduced reservoir with a depleted δ^13^C signature (32, 34–36, 47, 49, 54, 55). The regularity of the Early Eocene hyperthermals has drawn comparison with orbitally paced glaciation events of the Neogene, both in terms of the importance of orbital forcing and clear role of carbon cycle feedbacks (34). For the glacial–interglacial cycles of the Plio-Pleistocene, consensus has built around the importance of changes in ocean carbon storage to explain the atmospheric change in CO_2_, with changes in ocean CO_2_ uptake due to some unresolved combination of changes in the physical ocean circulation and the ocean’s biological carbon pump (1, 12–15, 56). A great deal of study has focused on changes in large scale overturning circulation between glacials and interglacials, with clear differences in benthic δ^13^C gradients, though not necessarily differences in overall overturning strength (e.g., refs. 4 and 57–61). Evidence for changes in overturning circulation across repeated Eocene hyperthermals raises the possibility that changes in ocean carbon storage may have acted as a carbon cycle feedback. Moreover, the presence of obliquity power in benthic δ^13^C records may provide further indication for the significance of changes in high latitude deepwater formation during Eocene hyperthermals. In our model experiment where we imposed the radiative forcing equivalent to a −1‰ δ^13^C excursion but without CO_2_ emissions, the resulting change in temperature and global overturning circulation led to an increase of atmospheric CO_2_ by 33 ppm. However, our modeling does not include representation of all carbon reservoirs that could be sensitive to the modeled changes in circulation (e.g., methane hydrates), so this is a conservative estimate of the potential feedback strength.

## 4. Conclusions

The relative amplification of benthic δ^13^C excursions in the early Eocene equatorial Atlantic Ocean provides evidence for periodic weakening and shoaling of southern-sourced deep-water formation during hyperthermal events. Results of Earth system modeling experiments are consistent with the isotopic observations, with large-scale overturning circulation being sensitive to even a relatively modest warming of ~2 °C. Our results thus demonstrate that large-scale overturning circulation was sensitive to changes in climate even prior to the establishment of a permanent cryosphere (and thus the potential for changes in ice melt to modulate deep water formation) and hence support the likelihood of continued weakening of North Atlantic overturning in response to ongoing anthropogenic emissions (62). Further, our results illustrate how spatial patterns in the magnitude of benthic δ^13^C excursions as a consequence of changes in ocean circulation invalidate assumptions that a benthic δ^13^C excursion magnitude derived from an individual location can be used to constrain past carbon forcing. Indeed, we find that changes in ocean circulation could amplify the apparent benthic δ^13^C excursions size by 0.6‰—a 50% increase relative to the global atmospheric signal. Overestimating the magnitude of a δ^13^C excursion would then lead to an overestimate in carbon emissions and consequent underestimate of climate sensitivity. This underscores the importance of generating globally distributed benthic δ^13^C records to accurately capture global gradients in water mass age across past transient climatic events.

## Supplementary Material

Appendix 01 (PDF)

Dataset S01 (XLSX)

Dataset S02 (XLSX)

Dataset S03 (XLSX)

## Data Availability

The code for the version of the “muffin” release of the cGENIE Earth system model used in this paper, is tagged as v0.9.51, and is assigned a DOI: https://doi.org/10.5281/zenodo.11189394 (63). Configuration files for the specific experiments presented in the paper can be found in the directory: https://github.com/derpycode/cgenie.muffin/tree/master/genie-userconfigs/PUBS/published/KirtlandTurner_et_al.2024 (64). Details of the experiments, plus the command line needed to run each one, as well as instructions for viewing the experiment results reported in this study are given in the readme.txt file in that directory. All other configuration files and boundary conditions are provided as part of the code release. Complete model output for all experiments included in this paper is assigned a DOI: https://doi.org/10.5281/zenodo.11187820 (65). A manual detailing code installation, basic model configuration, tutorials covering various aspects of model configuration, experimental design, and output, plus the processing of results, is assigned a DOI: https://doi.org/10.5281/zenodo.7545814 (66). All other data are included in the manuscript and/or supporting information.
