# Supplementary material for: Sensitivity of ocean circulation to warming during the Early Eocene greenhouse
Source: Proc Natl Acad Sci U S A. 2024 Jun 3;121(24):e2311980121. doi: 10.1073/pnas.2311980121 (PMC11181020; doi:10.1073/pnas.2311980121)
Supplement: Supplementary file 1 — Appendix 01 (PDF) [file pnas.2311980121.sapp.pdf]

Supplemental Figures

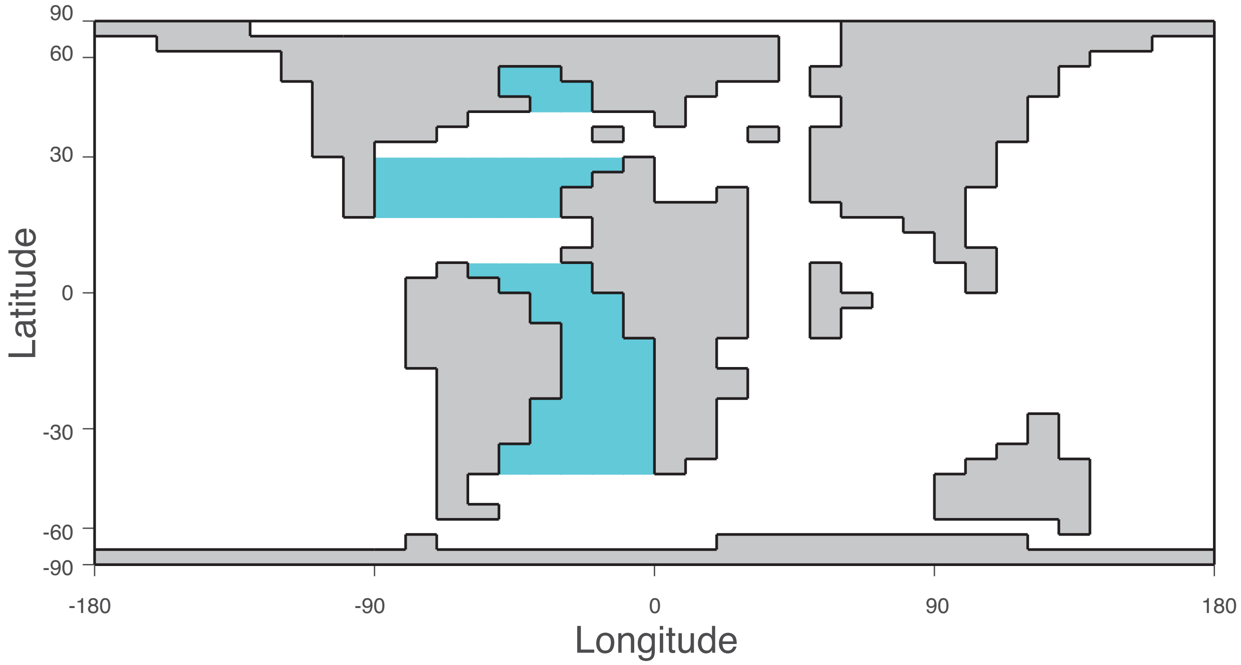

Supplemental Figure S1. cGENIE grid mask. The model resolution is 36x36 in the horizontal with grid spacing that is equal in longitude and the sine of latitude. The ocean has 16 vertical layers that exponentially increase in thickness with depth. The blue shaded region indicates the grid cells used to calculate the Atlantic Meridional Overturning streamfunction (plotted in Figure 4b). The streamfunction is only defined where there are zonal boundaries within the Atlantic.

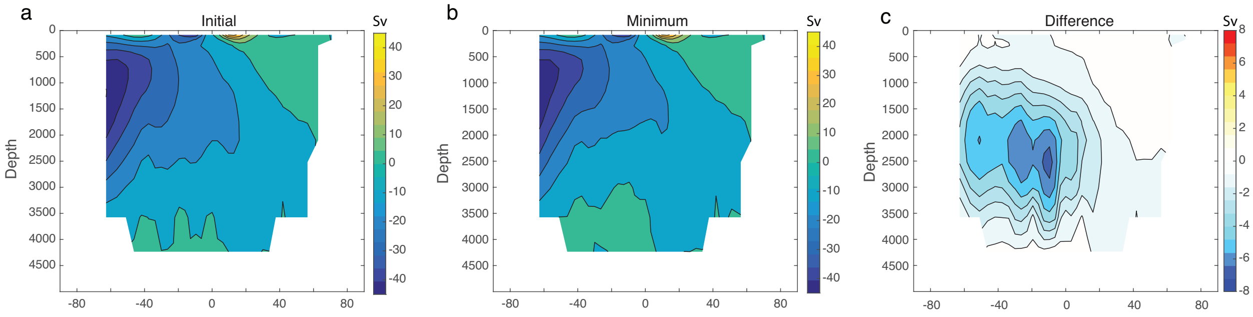

Supplemental Figure S2. cGENIE global meridional overturning streamfunction in the Early Eocene configuration. a) Initial (steady-state) streamfunction prior to perturbation. Overturning is dominated by the counter-clockwise circulation (driven by sinking in the Southern Ocean). Stronger counterclockwise overturning is indicated by darker blue colors while stronger clockwise overturning is indicated by yellow colors. b) Streamfunction from 6000 years into the modeled hyperthermal warming event when overturning is at minimum strength. c) Difference plot in the global meridional overturning streamfunction (initial - yr 6000). Weaker counter-clockwise overturning strength is plotted as blue colors.

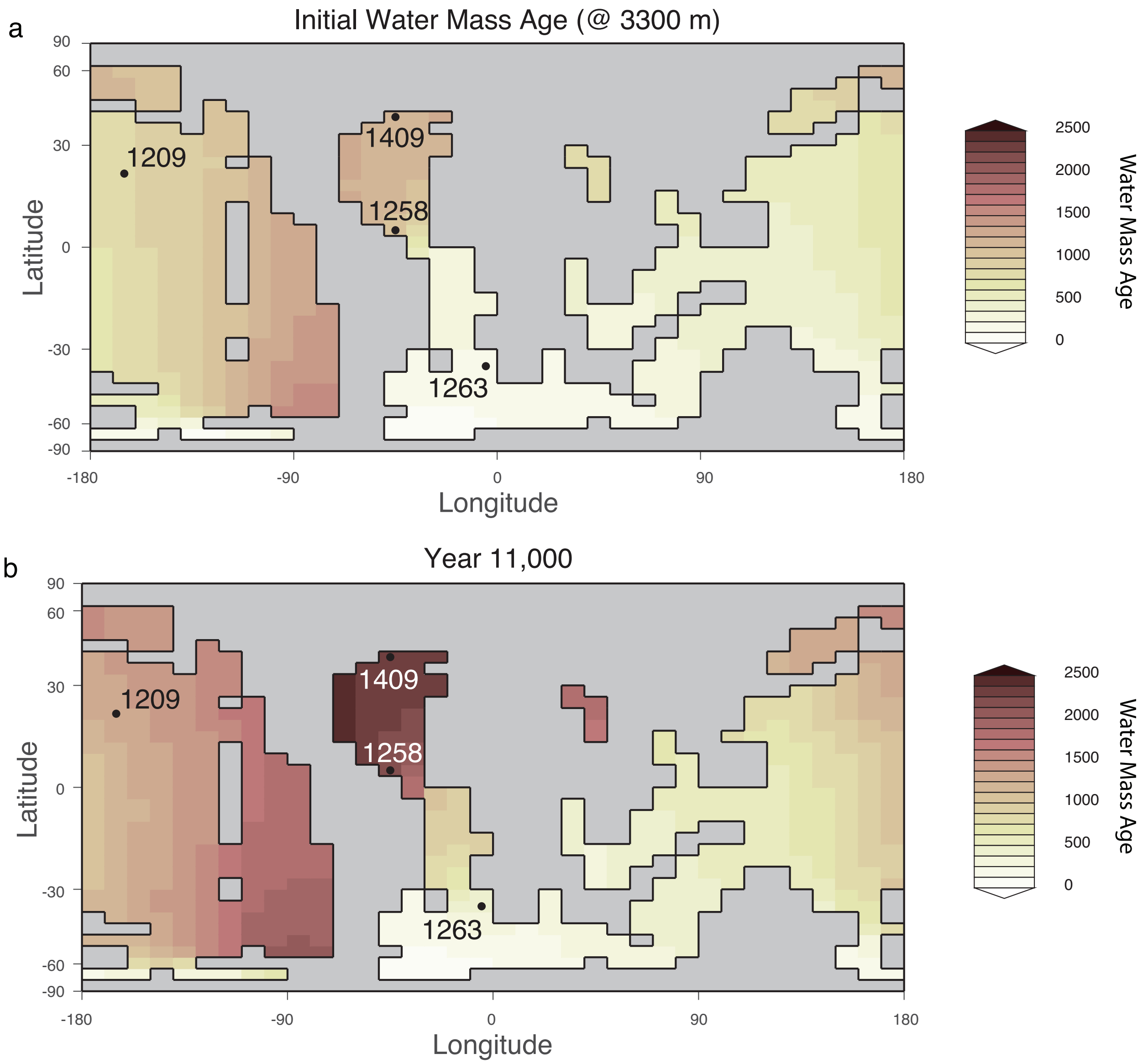

Supplemental Figure S3. cGENIE modeled ventilation ages at the modeled ocean depth layer with a midpoint of ~3300 m. a) Initial water mass age distribution prior to perturbation. b) Water mass age distribution from 11000 years into the modeled hyperthermal warming vent when aging gradients are a maximum. Note: difference map is plotted in Figure 4c.

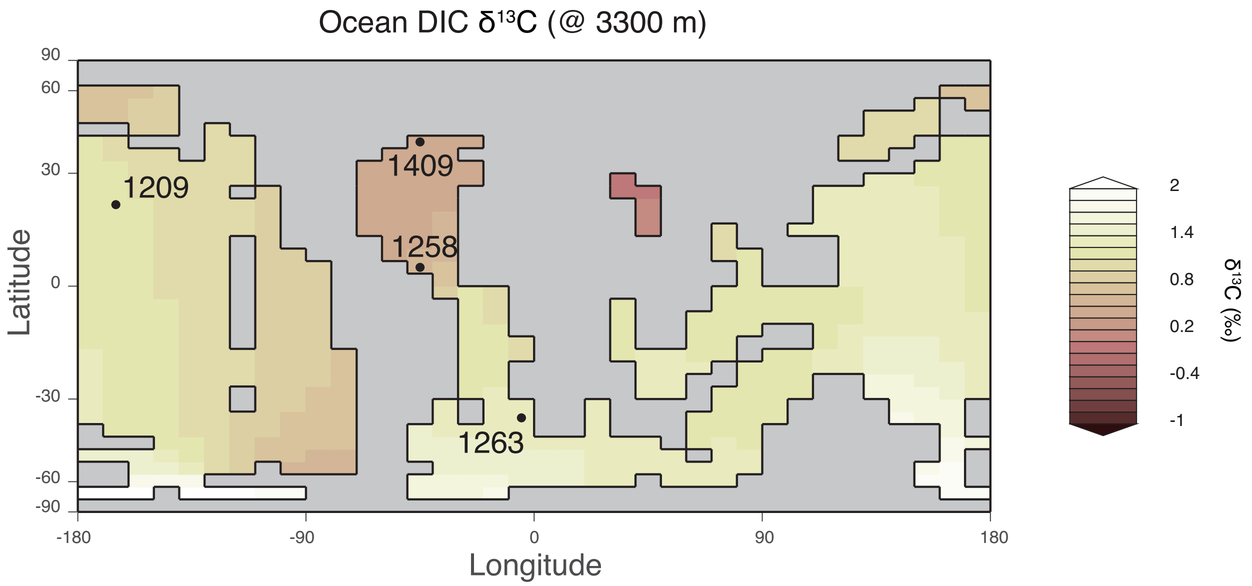

Supplemental Figure S4. cGENIE Early Eocene modeled deep ocean $\delta^{13}C$ of dissolved inorganic carbon at the ocean depth layer with a midpoint of ~3300 m.

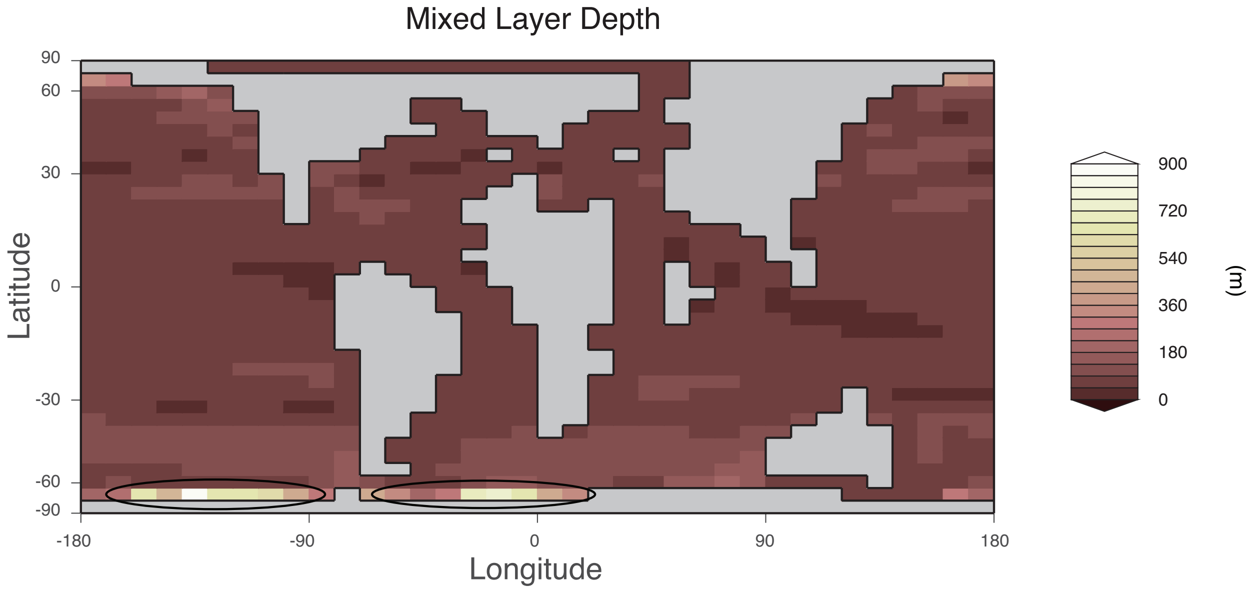

Supplemental Figure S5. cGENIE Early Eocene mixed layer depth, which indicates areas of deep convection. Circles indicate areas of deep convection, which are mainly in the Southern Ocean in the South Atlantic and South Pacific. Another region of less significant deep convection occurs in the North Pacific.

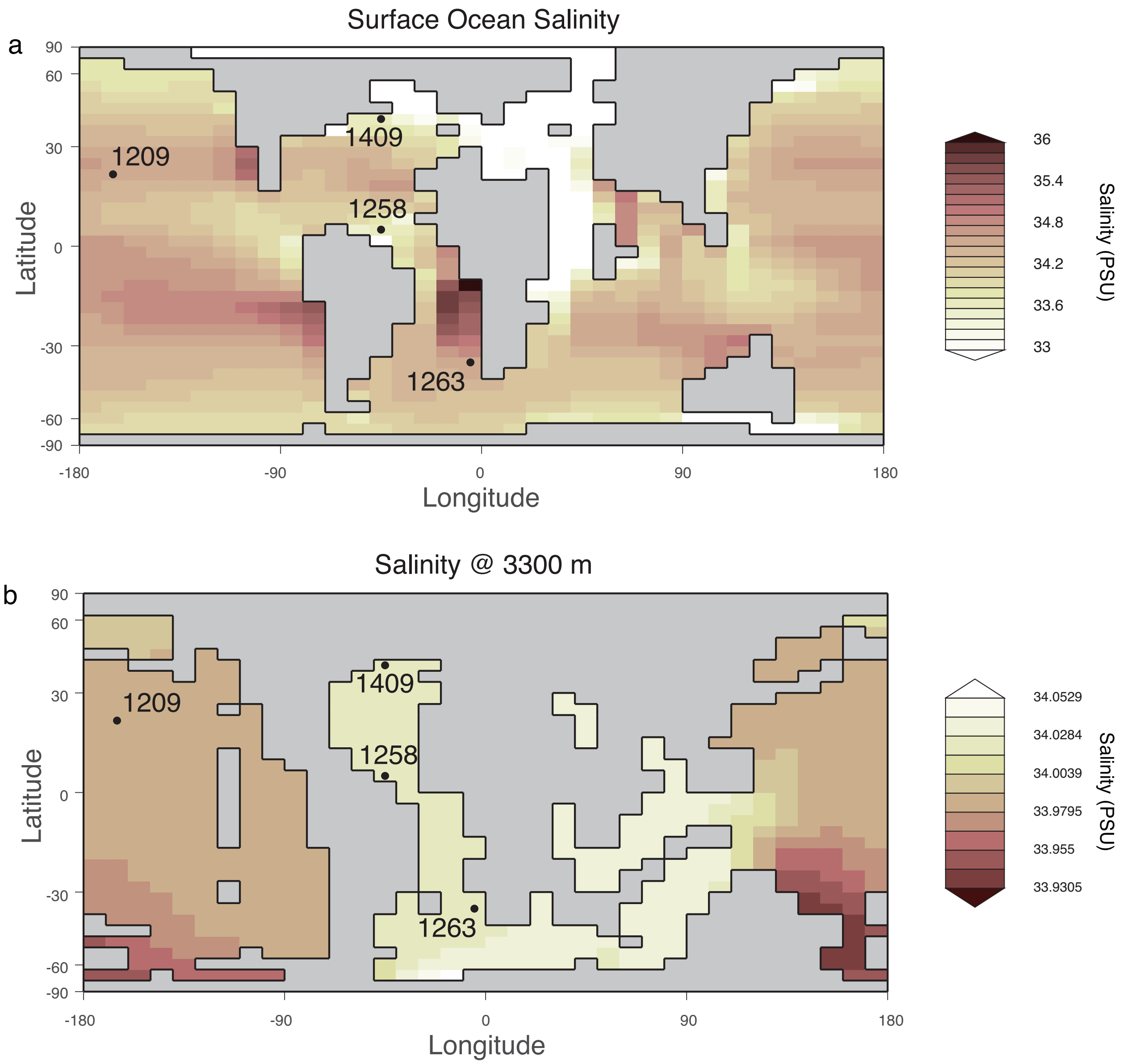

Supplemental Figure S6. cGENIE Early Eocene salinity distribution. a) Sea surface salinity. b) Salinity at the ocean depth layer with a midpoint of ~3300 m. Note change in colorbar scale between plots a) and b) (salinity variations are much less at depth).

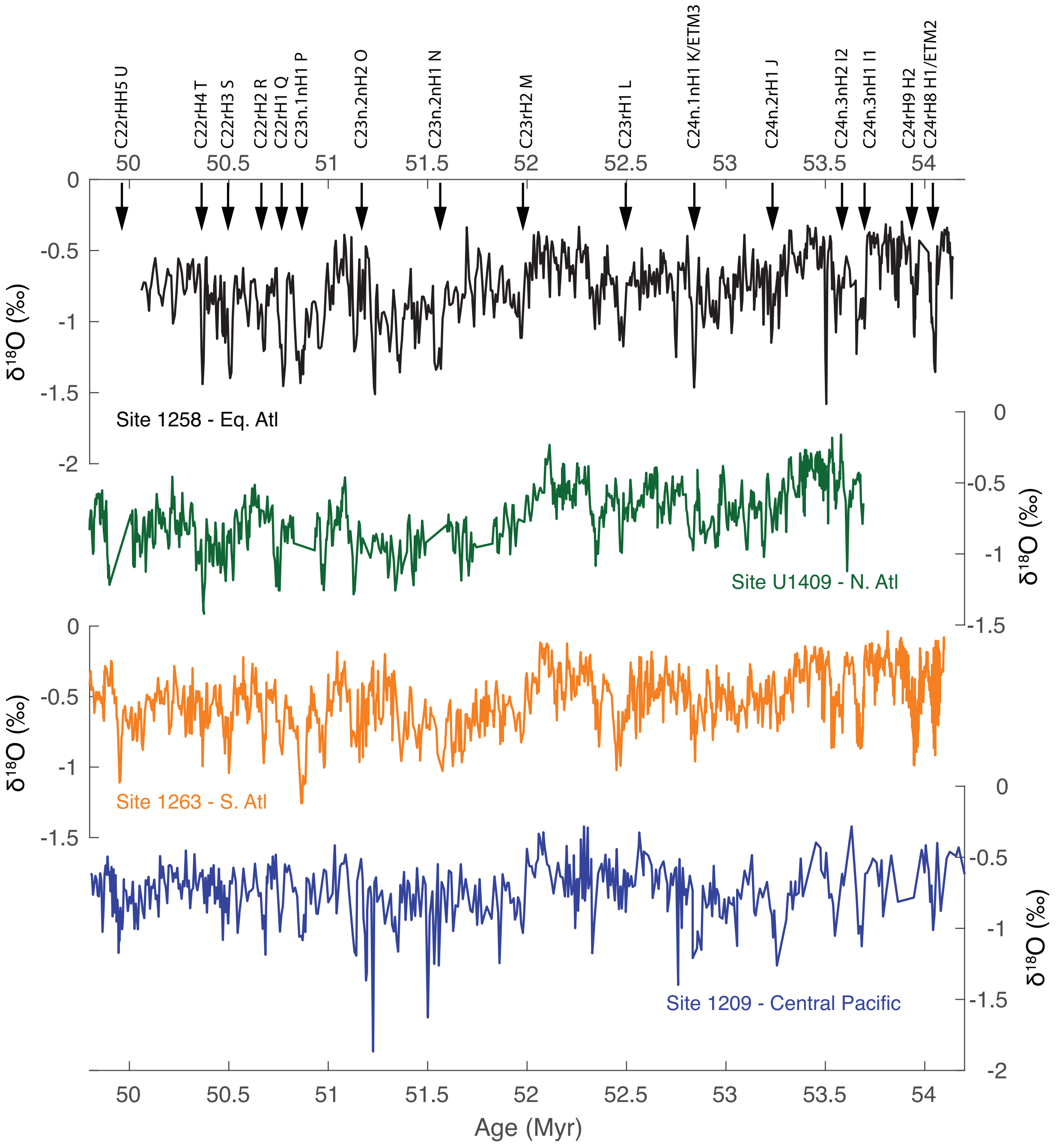

Supplemental Figure S7. Benthic foraminiferal $\delta^{18}O$ records from 4 sites across the Early Eocene Climate Optimum, from top to bottom: ODP Site 1258 in the equatorial Atlantic, IODP Site U1409 in the North Atlantic, ODP Site 1263 in the South Atlantic, and ODP Site 1209 in the Central Pacific. Labeled arrows indicate hyperthermal events identified from Site 1209 by Westerhold et al., 2018.

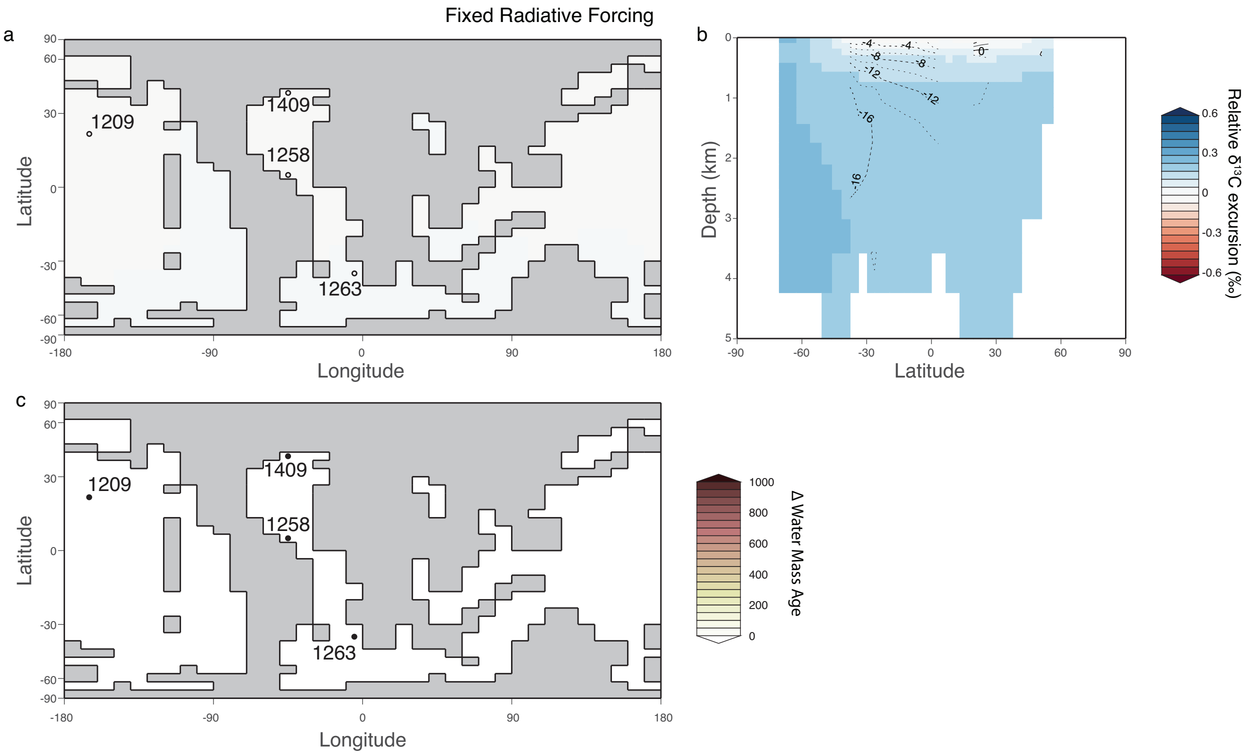

Supplemental Figure S8. Spatial patterns in cGENIE DIC $\delta^{13}C$ excursion size and water mass age in response to 1‰ CIE with fixed radiative forcing (i.e. no warming or circulation response). a) Relative size of the $\delta^{13}C$ excursion recorded for each model grid relative to the mean $\delta^{13}C$ excursion in the ocean layer centered at ~3300 m water depth. $\delta^{13}C$ excursions are calculated as the DIC $\delta^{13}C$ value at 11 kyr less the initial value in that grid. Red colors (more negative) indicate CIEs larger than the average. b) Atlantic zonal mean size of the $\delta^{13}C$ excursion relative to the atmospheric $\delta^{13}C$ excursion. $\delta^{13}C$ excursions are calculated as the DIC $\delta^{13}C$ value at 11 kyr less the initial value. Contours show the Atlantic Meridional Overturning streamfunction at 11 kyr. c) Relative size of the change in water mass age recorded for each model grid relative to the mean change in water mass age in the ocean layer centered at ~3300 m water depth. Changes in water mass age are calculated as the water mass age at 11 kyr less the initial age in that grid. Note that subplots (a) and (c) show white (a value of zero everywhere), which indicates that there are no patterns in either the size of the $\delta^{13}C$ excursion or change in water mass age, i.e. every grid cell shows the same change.

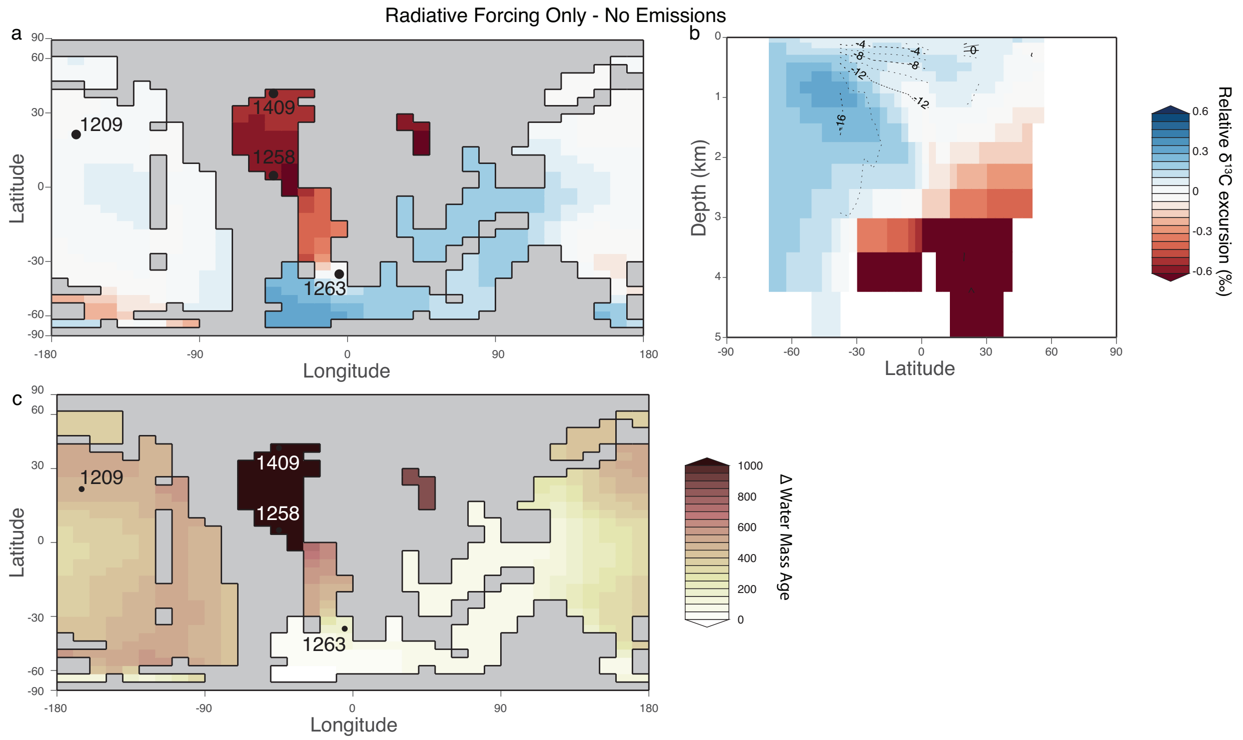

Supplemental Figure S9. Spatial patterns in cGENIE DIC $\delta^{13}C$ excursion size and water mass age in response to radiative forcing equivalent to the 1‰ CIE experiment but without CO_2 emissions. Because this experiment has radiative forcing imposed without carbon emissions, the change in atmospheric and mean ocean $\delta^{13}C$ is very small (see Supplemental Figure S5a). a) Relative size of the $\delta^{13}C$ excursion recorded for each model grid relative to the mean $\delta^{13}C$ excursion in the ocean layer centered at ~3300 m water depth. $\delta^{13}C$ excursions are calculated as the DIC $\delta^{13}C$ value at 11 kyr less the initial value in that grid. Red colors (more negative) indicate CIEs larger than the average. b) Atlantic zonal mean size of the $\delta^{13}C$ excursion relative to the atmospheric $\delta^{13}C$ excursion. $\delta^{13}C$ excursions are calculated as the DIC $\delta^{13}C$ value at 11 kyr less the initial value. Contours show the Atlantic Meridional Overturning streamfunction at 11 kyr. c) Relative size of the change in water mass age recorded for each model grid relative to the mean change in water mass age in the ocean layer centered at ~3300 m water depth. Changes in water mass age are calculated as the water mass age at 11 kyr less the initial age in that grid. Red/burgundy colors indicate larger increases in water mass age than the global average.

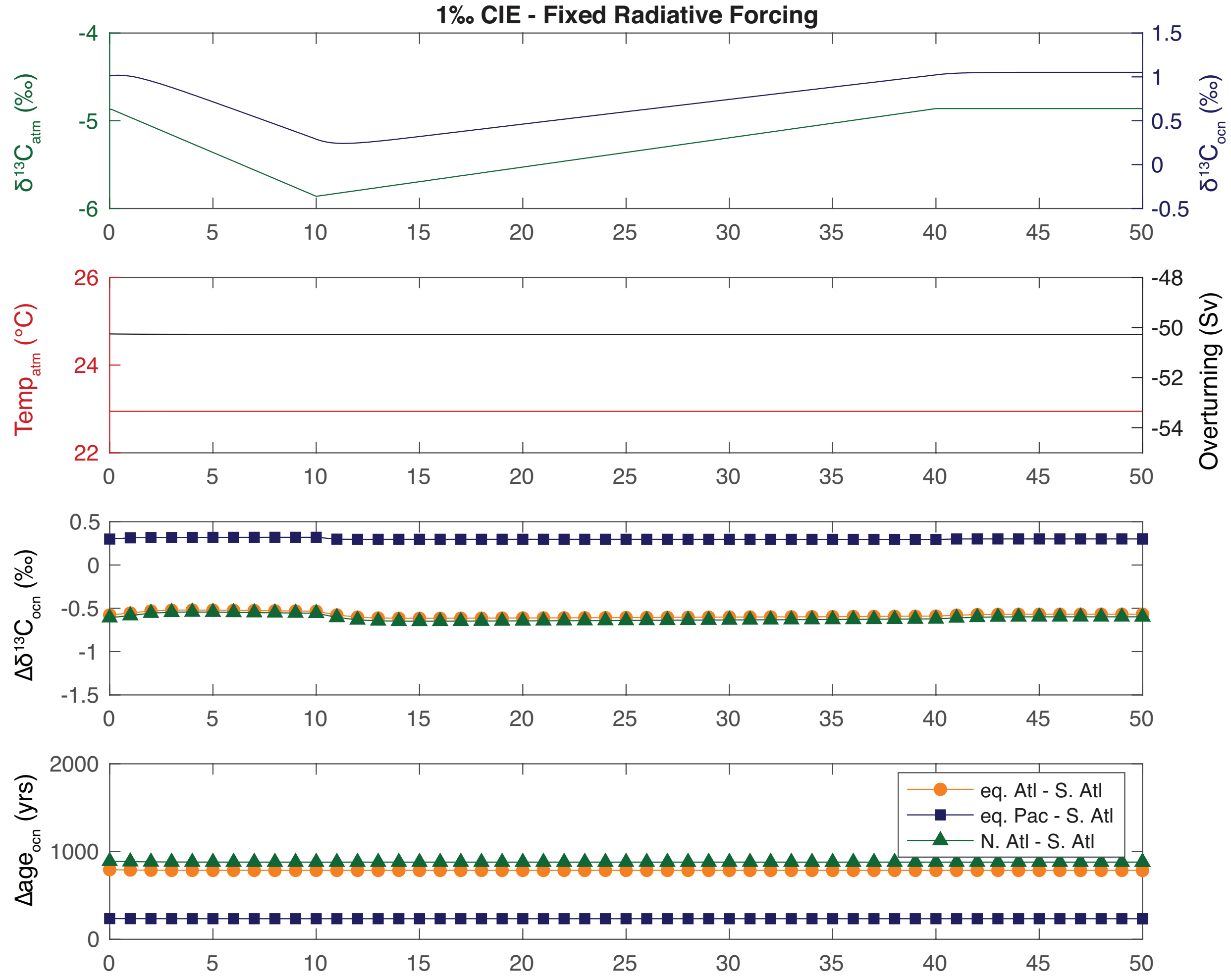

Supplemental Figure S10. cGENIE model output of changes in climate and ocean circulation in response to a 1‰ CIE with fixed radiative forcing. a) Atmospheric $\delta^{13}C$ (green) and benthic ocean DIC $\delta^{13}C$ (blue). b) Atmospheric temperature (red) and counter-clockwise (Southern-sourced) overturning streamfunction (black). c) Change in the deep ocean DIC $\delta^{13}C$ gradient between the equatorial Atlantic and South Atlantic (orange), the North Atlantic and South Atlantic (green) and between the equatorial Pacific and South Atlantic (blue). d) Changes in water mass aging gradients between the equatorial Atlantic and South Atlantic (orange), the North Atlantic and South Atlantic (green) and between the equatorial Pacific and South Atlantic (blue).

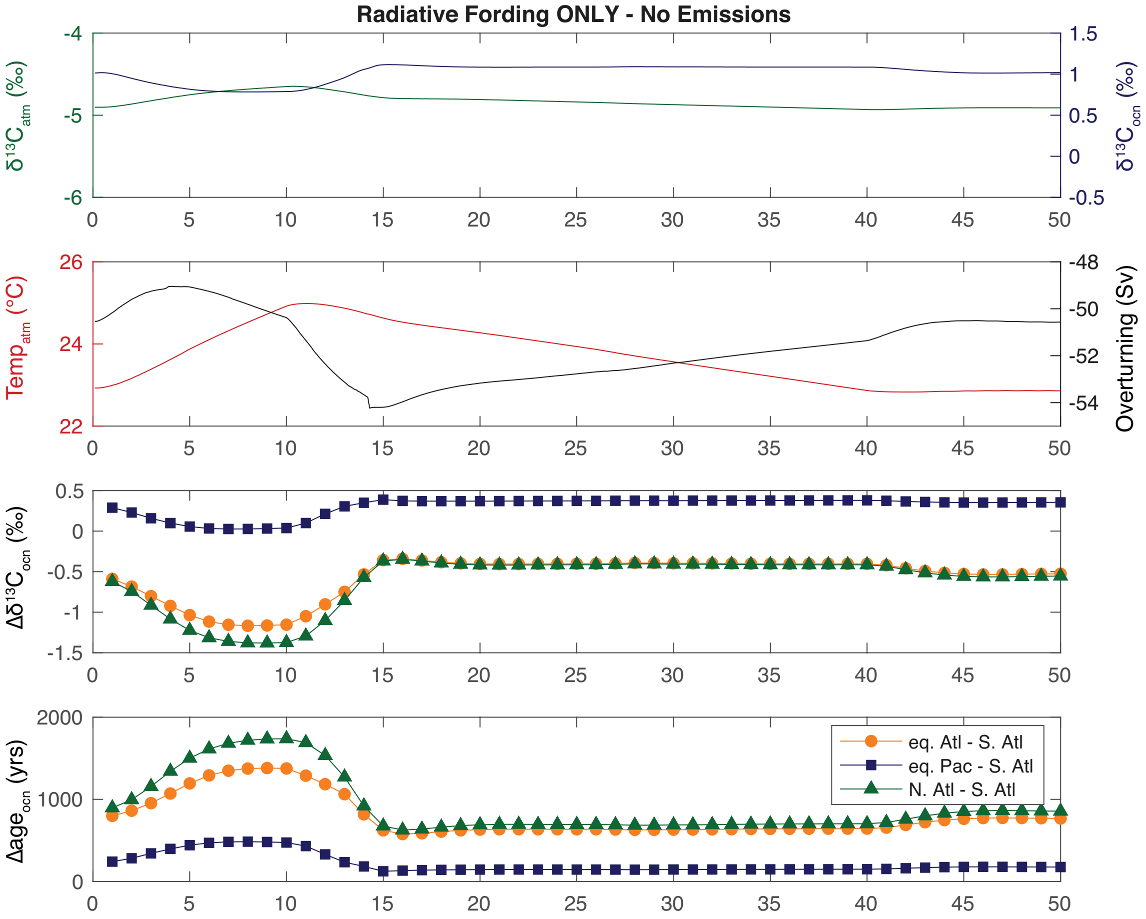

Supplemental Figure S11. cGENIE model output of changes in climate and ocean circulation in response to radiative forcing equivalent to the 1‰ CIE experiment but without CO_2 emissions. a) Atmospheric $\delta^{13}C$ (green) and benthic ocean DIC $\delta^{13}C$ (blue). b) Atmospheric temperature (red) and counter-clockwise (Southern-sourced) overturning streamfunction (black). c) Change in the deep ocean DIC $\delta^{13}C$ gradient between the equatorial Atlantic and South Atlantic (orange), the North Atlantic and South Atlantic (green) and between the equatorial Pacific and South Atlantic (blue). d) Changes in water mass aging gradients between the equatorial Atlantic and South Atlantic (orange), the North Atlantic and South Atlantic (green) and between the equatorial Pacific and South Atlantic (blue).

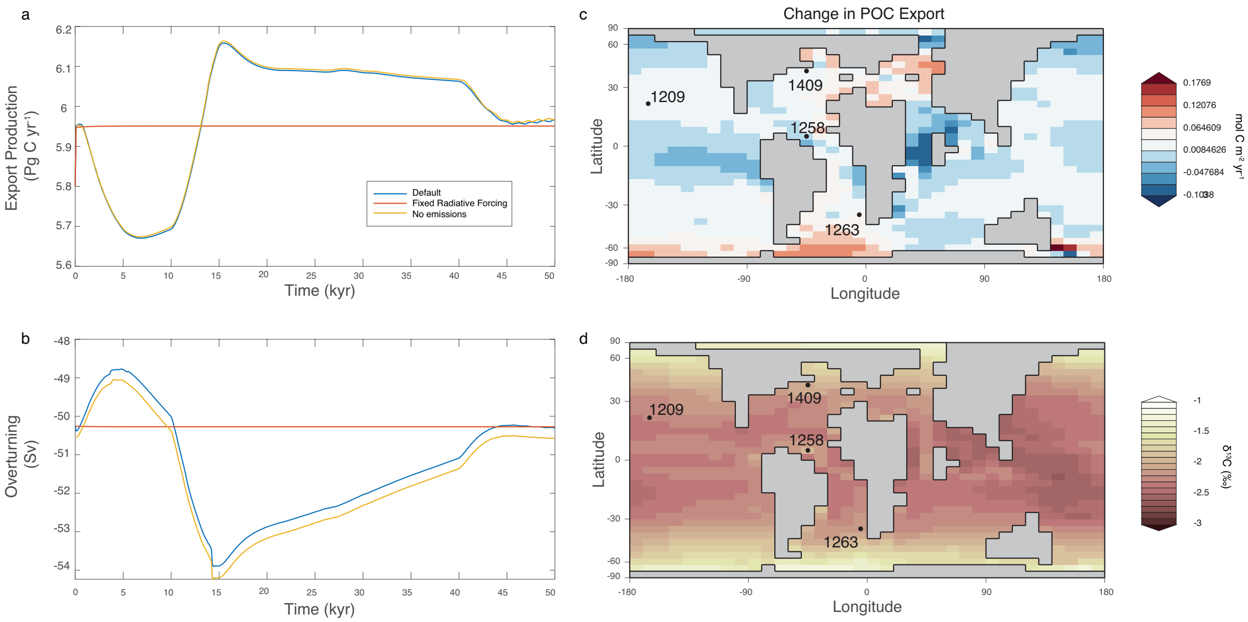

Supplemental Figure S12. Modeled changes in export production in response to hyperthermal warming in cGENIE. a) Change in total export production in all three experiments in Pg per year - blue is the default experiment where injection of isotopically depleted CO_2 leads to warming, orange is the fixed radiative forcing case with disabled CO_2-climate feedback, and yellow is the experiment with diagnosed radiative forcing but no CO_2 emissions. b) Change in counter-clockwise global meridional overturning circulation in all three experiments. c) Change in POC export (Yr 11,000 - initial) in response to hyperthermal warming. Red colors indicate regions of enhanced POC export. d) Change in the $\delta^{13}C$ of POC export (Yr 11,000 - initial) indicating the surface $\delta^{13}C$ patterns that are transmitted to the deep ocean via the biological carbon pump. At the high latitudes, the change in the $\delta^{13}C$ of POC export is less pronounced compared to the mid to low latitudes.
